# Collision-Induced Dissociation of Fucose and Identification of Anomericity

**DOI:** 10.1021/acs.jpca.4c00640

**Published:** 2024-05-01

**Authors:** Hock-Seng Nguan, Jien-Lian Chen, Chi-Kung Ni

**Affiliations:** † Institute of Atomic and Molecular Sciences, Academia Sinica, P.O. Box 23-166, Taipei 10617, Taiwan; ‡ Department of Chemistry, National Tsing Hua University, Hsinchu 30013, Taiwan

## Abstract

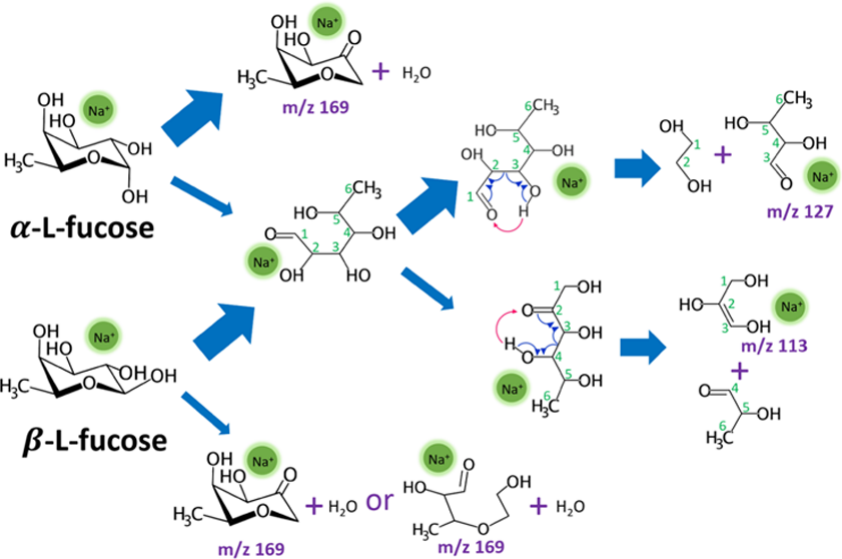

Structural determination of carbohydrates using mass spectrometry remains challenging, particularly, the differentiation of anomeric configurations. In this work, we studied the collision-induced dissociation (CID) mechanisms of sodiated α- and β-l-fucose using an experimental method and quantum chemistry calculations. The calculations show that α-l-fucose is more likely to undergo dehydration due to the fact that O1 and O2 are on the same side of the sugar ring. In contrast, β-l-fucose is more prone to the ring-opening reaction because more OH groups are on the same side of the sugar ring as O1. These differences suggest a higher preference for the dehydration reaction in sodiated α-l-fucose but a lower preference for ring-opening compared to that of β-l-fucose. The calculation results, which are used to assign the CID mass spectra of α- and β-l-fucose separated by high-performance liquid chromatography, are supported by the fucose produced from the CID of disaccharides Fuc-β-(1 → 3)-GlcNAc and Fuc-α-(1 → 4)-GlcNAc. This study demonstrates that the correlation of cis- and trans-configurations of O1 and O2 to the relative branching ratios of dehydration and cross-ring dissociation in CID, observed in aldohexose and ketohexose in the pyranose form, can be extended to deoxyhexoses for anomericity determination.

## Introduction

**Figure 1:**
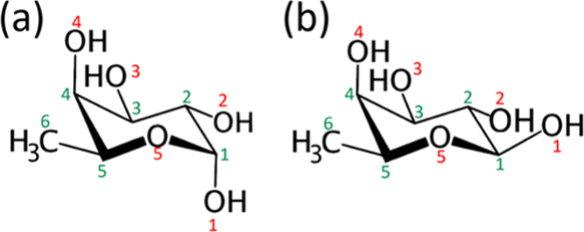
α-l-fucose (a) and β-l-fucose (b) in the chair form. The numberings of oxygen and carbon atoms are given by the numbers in red and green, respectively.

Fucose is a deoxyhexose with five hydroxyl groups (see Figure 1). Many glycans in mammals contain l-fucose,^1,2^ and fucose plays an important role in numerous biological processes. For example, studies have shown that the presence of fucose in *N*-glycan can be related to inflammation, immune responses, and cancer metastasis.^1,3−8^ Another example is the fucosylated oligosaccharides in milk. Many fucosylated oligosaccharides have been found in human milk, and they act as prebiotics, deflect the adhesion of pathogens in the infant gut, and affect the local intestinal immune system of breastfed infants.^9−15^

Understanding the biological functions of these fucose-containing glycans necessitates characterizing their structures. Traditional methods for glycan structural determination involve nuclear magnetic resonance spectroscopy^16,17^ and enzyme digestion.^18−20^ However, characterizing these structures is challenging, partly because obtaining them in large quantities for NMR and enzyme digestion is difficult and partly because there is a lack of suitable enzymes for differentiating among structures. Tandem mass spectrometry has become widely used in glycan structural determination due to the high sensitivity of mass spectrometry.^21−30^ In tandem mass spectrometry, glycans are dissociated into fragments, and glycan structures are inferred by the mass spectra of these fragments. Conventional tandem mass spectrometry methods typically determine the linkage positions of carbohydrates, yet the differentiating anomericity, such as α-fucose and β-fucose, remains challenging. When analyzing fucosylated glycans, tandem mass spectrometry should be used with caution as fucose may migrate from one position to another position of glycans upon activation during fragmentation,^31,32^ leading to incorrect linkage assignments.^33^ The undesirable fucose migration occurs easily in protonated glycan ions, whereas in sodiated glycan ions, i.e., glycan + Na^+^ complexes, it does not occur. Recently developed techniques of ion mobility^34,35^ and infrared action spectra^36−38^ were shown to be able to differentiate some carbohydrate anomericities according to their ion mobility differences and spectrum difference, respectively. Another recent approach that can characterize anomericity has been proposed by us,^39^ which is based on conventional tandem mass spectrometry with the understanding of the dissociation mechanism through quantum chemistry calculations.

Quantum chemistry calculations provide useful information regarding the carbohydrate dissociation behaviors in mass spectrometry. Since the 1990s, a series of theoretical calculations have been carried out using the semiempirical Hartree–Fock method to study the conformations or dissociation of disaccharides^40−43^ and even larger carbohydrates during collision-induced dissociation (CID).^44,45^ Recently, more accurate methods like density functional theory (DFT) have been used to investigate monosaccharide and disaccharide CID.^46−57^ High-level quantum chemistry calculations yield more accurate dissociation barrier heights and transition-state structures, aiding in the elucidation of dissociation mechanisms and the explanation of fragments produced during CID. However, sugars are floppy molecules; sugar rings can assume various ring-puckering forms, and H atoms of their OH functional groups can easily change orientation. Consequently, there are over 200 different conformers for a hexose monosaccharide.^58^ When two monosaccharides are linked, the two dihedral angles of the glycosidic bond can easily change, resulting in over a million disaccharide conformers.^55,57^ Calculating only a handful of conformers may not accurately predict the mass spectra since the transition state (TS) with the lowest barrier height is not necessarily correlated to the most stable conformer. An efficient method to search for low-lying TSs is essential for understanding the dissociation mechanisms of monosaccharides and disaccharides in the CID. Recently, we have developed an effective method to search for conformations and low-lying TSs of monosaccharides and disaccharides attached to a sodium ion. Our previous computational studies of monosaccharides^46,49,53,54,59^ and disaccharides^55−57^ have enabled the identification of major dissociation pathways, thereby facilitating the correct interpretation of fragmentation patterns observed in the mass spectra. Applying these dissociation mechanisms to oligosaccharides enables the correct assignment of oligosaccharide structures.^39,60−63^

In this work, we investigate the CID mechanism of α- and β-l-fucose using experimental methods and theoretical calculations. We conduct CID experiments on fucose monosaccharide and fucose generated from the dissociation of fucose-containing disaccharides. To understand the detailed dissociation mechanism, molecular modeling and quantum chemistry calculations of l-fucose monosaccharide are conducted to identify as many low-lying TSs as possible for major reaction channels in CID. These TSs and their corresponding reactant states, obtained from quantum chemistry calculations, are utilized to interpret the experimental results. We show that α- and β-anomers of fucose, which are very difficult to distinguish using conventional mass spectrometry methods, can be readily differentiated using the CID of sodiated fucose.

## Experimental Method

Fucose and disaccharides Fuc-β-(1 → 3)-GlcNAc and Fuc-α-(1 → 4)-GlcNAc were purchased from Omicron Biochemicals, Inc. (South Bend, IN). The purity of these compounds was checked by using high-performance liquid chromatography (HPLC). For HPLC-electrospray ionization (ESI)-MS experiments, 5 μL of the sample, dissolved in deionized water (DIW) at a concentration of 2 × 10^–4^ M, was injected into the HPLC system (Dionex Ultimate 3000, Thermo Fisher Scientific Inc., Waltham, MA) with a Hypercarb column (100 × 2.1 mm^2^, particle size: 3 μm, Thermo Fisher Scientific Inc.) for the separation of anomers. The eluents were then injected into an ESI source of a linear ion trap mass spectrometer (LTQ XL, Thermo Fisher Scientific Inc.). A NaCl solution (2 × 10^–4^ M NaCl dissolved in methanol/DIW = 50:50 solution (v/v%)) was added to the HPLC eluents before entering the ESI source. The mobile phase of the HPLC consisted of DI water (solution A) and HPLC-grade acetonitrile (solution B). The gradient of the mobile phase was changed linearly from A = 100% and B = 0% at *t* = 0 min to A = 90% and B = 10% at *t* = 30 min, with a flow rate of 200 μL/min. The temperatures of the ESI source and transfer capillary were set at 280 and 350 °C, respectively. The voltages of the ion spray, capillary, and tube lens were set at 4000, 80, and 150 V, respectively. Helium gas was used as the buffer gas and the collision gas used in the ion trap. The linear ion trap settings were an isolation width of 1 μ, activation time of 30 ms, normalized collision energy of 30%, and a Q value of 0.25 in the positive mode. For ESI-MS experiments, the analytes were dissolved in a mixture of methanol/DIW (v/v% 50:50) solution at a concentration of 2 × 10^–4^ M with NaCl of 2 × 10^–4^ M. The temperature of the ESI source was set to 50 °C. The other experimental parameters were the same as those used for the HPLC-ESI mass spectrometry experiment.

## Computational Method

**Figure 2:**
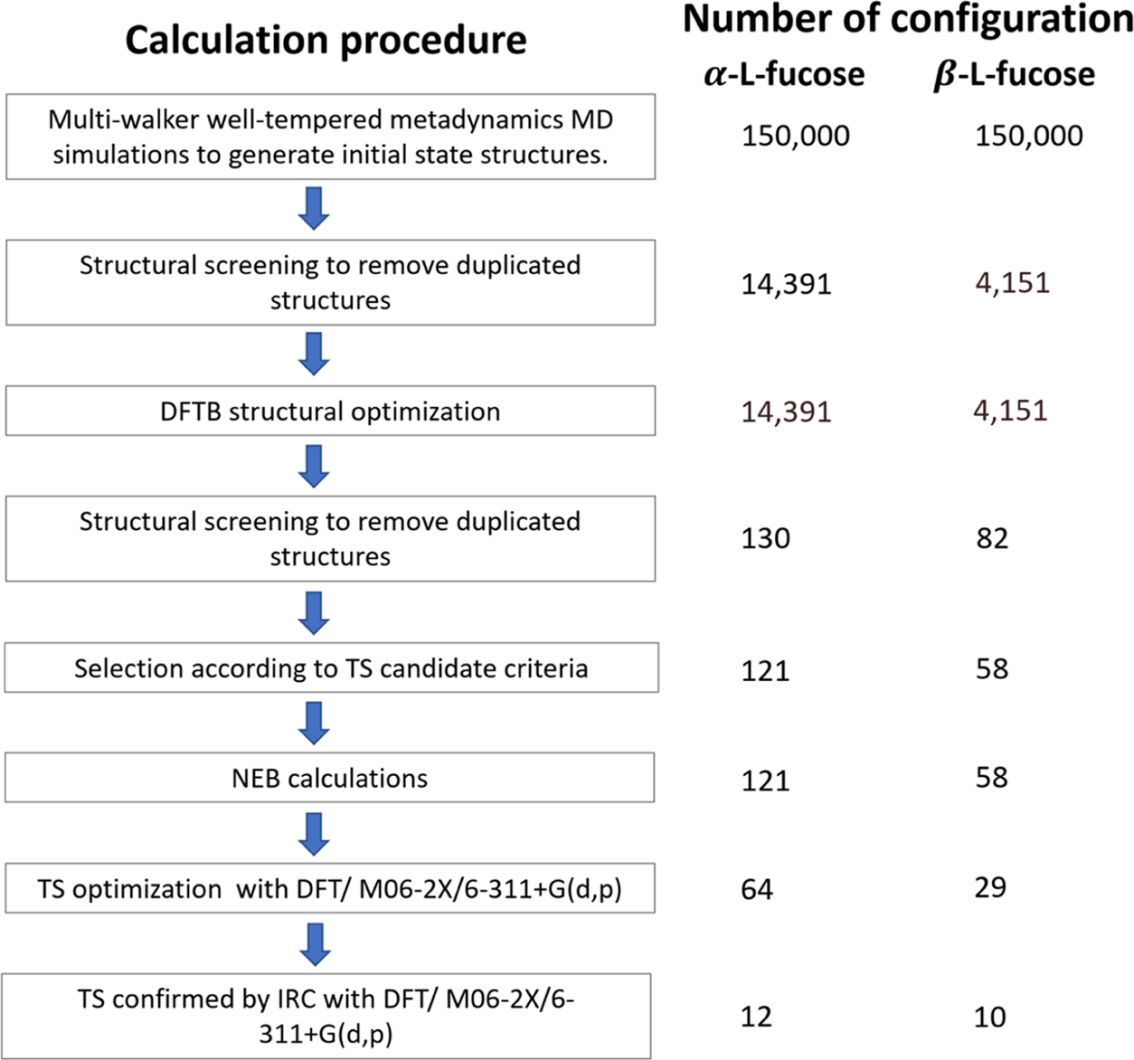
Calculation procedure and number of configurations involved in each stage of calculations.

Figure 2 illustrates our computational procedure, detailing the calculation methods at each stage and the number of structures involved or obtained in each stage of the calculations. The procedure initiates with a conformational search to identify as many low-energy conformers as possible, which serve as initial conformers for finding low-lying TSs for the reactions of interest. Our previous studies of hexose and N-acetylhexsamine^46,49,53,54,59^ suggest that the sodium ion’s position plays a crucial role in the CID of fucose, which primarily involves dehydration and cross-ring dissociation. In the initial stage, we employed metadynamic molecular dynamics (MD) simulations to explore various conformers of l-fucose + Na^+^ complexes in a vacuum. For both sodiated α- and β-l-fucose, we conducted three multiwalker well-tempered metadynamics simulations, applying 10 walkers to each MD simulation. Bias forces were introduced in the metadynamic MD simulations to facilitate the sampling of broader conformational spaces, implemented through the settings of the collective variables. We used two collective variables: the Cremer–Pople puckering index^64^ and the sodium ion to O atom coordination number. The former helped in sampling various ring-puckering forms of fucose, while the latter helped in exploring different sodium ion coordinations to the O atoms. The three simulations have different rates of bias energy additions—0.001 0.002, and 0.01 hartree—to yield a more diverse range of structures. The forces and the energy in the simulations were computed using density functional theory tight-binding (DFTB).^65^

Conformers resulting from the simulations were screened using the Ballester and Richards^66^ method. Those with similarity scores above 95% were grouped together, and only the lowest-energy conformer from each group was selected for further calculations. These were then geometrically optimized using the DFTB method and were screened once again with the same criteria. The resulting structures were analyzed to identify potential reactant states leading to the low-lying TSs based on the premise from our previous computational studies^46,57^ that the binding of the sodium ion can weaken the O–H or O–C bonds of the associated O atom, thus lowering the TS energy. For the dehydration reaction, we focused on the H atom transfer to the O1 atom and the C1–O1 cleavage, as the OH group at C1 is highly reactive due to the hemiacetal functional group’s nature. Cross-ring dissociation begins with a ring-opening reaction, which transfers the H atom from the O1 atom to the O5 atom. Since the ring-opening reaction is the rate-determining step in the series of reactions leading to cross-ring dissociation,^46^ our calculations for cross-ring dissociation only consider the ring-opening reaction. The criteria for a reactant state leading to low-lying TSs are (1) the sodium ion must bind to at least one O atom with a Na^+^–O distance of less than 2.5 Å,^46,49^ ensuring the reactant energy is not too high; (2) for the reaction of dehydration, if the sodium ion binds to O2, O3, or O4, the distance between the O1 atom and the binding O atom (O2, O3, or O4) should be less than 3 Å, indicating a lower barrier of H atom transfer; and (3) for the ring-opening reaction, if the sodium ion binds to O1 or O5, the Na^+^–O1 or Na^+^–O5 distance must be less than 2.5 Å,^46,49^ facilitating the weakening the O1–H and O5–C1 bonds and thus reducing the reaction barrier height.

Subsequently, we performed climbing-image nudge-elastic band (NEB)^67^ calculations on the chosen conformers as the reactant candidates to obtain the reaction pathways as well as the TSs of the reactions. These calculations employed the DFTB method, and the resulting TSs were used as initial guesses for more precise TS optimization via the DFT method. The DFT-optimized TSs were further verified by intrinsic reaction coordinate^68^ (IRC) calculations. All metadynamic simulations, DFTB structure optimizations, and NEB calculations were carried out using CP2K software (version 5.1).^69^ The DFTB method employed was DFTB3,^65,70,71^ with the third-order parametrization for organic and biological system (3OB)^72^ parameters. The DFT calculations utilized Gaussian 16^73^ with the M06-2*X*/6-311+(d,p) level of theory.

## Results and Discussion

**Figure 3:**
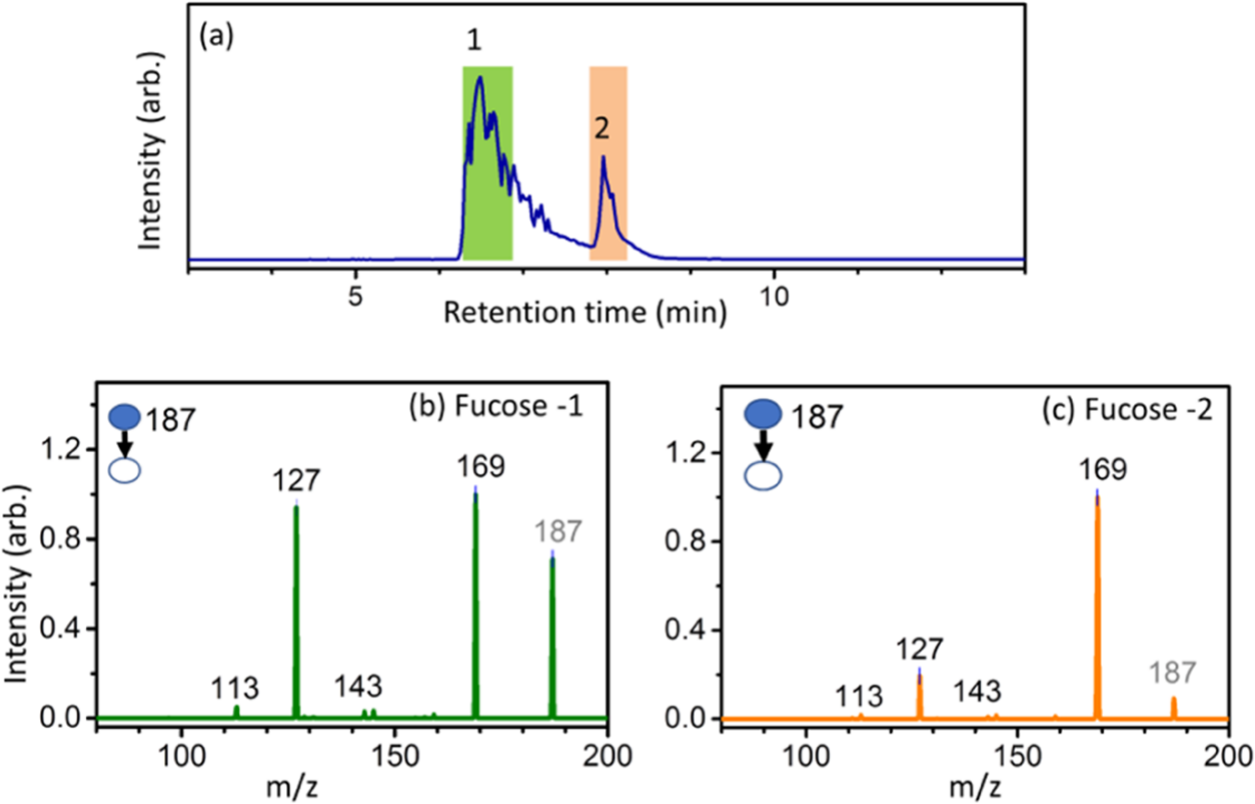
Chromatogram of l-fucose (a) and CID mass spectra of peak 1 (b) and peak 2 (c). The mass spectra in green and orange were measured during the retention time highlighted in green and orange. The collision energy is 30% of the normalized collision energy. The relative intensities of fragments in the spectra do not change in the collision energy region from 30 to 50%.

The α- and β-anomers of fucose coexist in solution and reach equilibrium through mutarotation. The mutarotation typically takes 30 min to a couple of hours to reach equilibrium, depending on the solvent and temperature. There are two peaks in the HPLC chromatogram of fucose if these two anomers are separated by HPLC within the time before they change from one to the other. Figure 3 shows the chromatogram and the corresponding CID mass spectra of fucose. The two peaks in the chromatogram represent two anomers of fucose separated by HPLC. In the mass spectra, the precursor ion *m*/*z* 187 represents the sodium ion adduct of fucose, and ions *m*/*z* 169 and 127 represent the product ions of dehydration and cross-ring dissociation, respectively. The spectra show a large intensity ratio of ion *m*/*z* 169 to ion *m*/*z* 127 for the peak at retention time 8.0 min, while the intensity of ion *m*/*z* 169 is similar to that of ion *m*/*z* 127 for the peak at retention time 6.5 min. However, the assignment of α- and β-anomers to these two peaks requires additional information.

**Figure 4:**
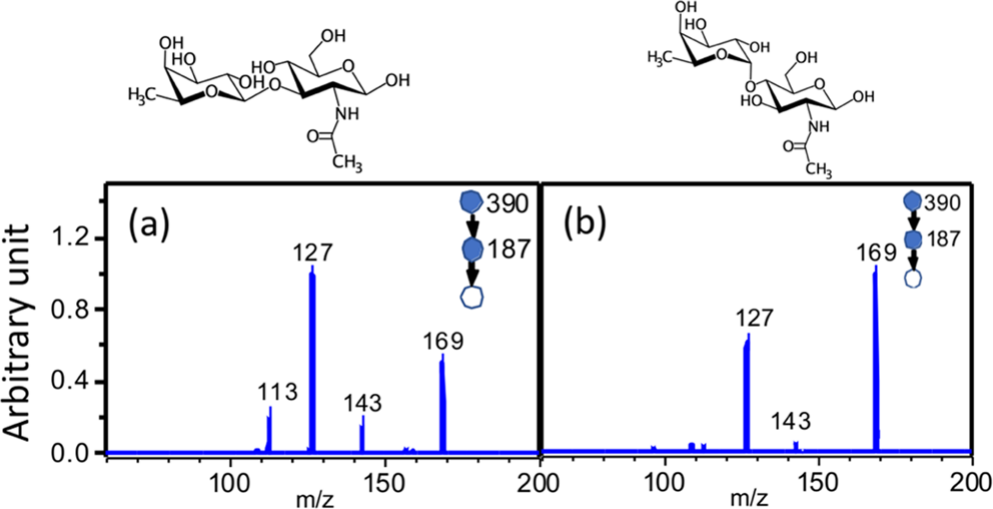
CID mass spectra of the fucose generated from disaccharides Fuc-β-(1 → 3)-GlcNAc (a) and Fuc-α-(1 → 4)-GlcNAc (b) through the CID sequence 390 → 187 →.

Figure 4a,b show the CID mass spectra of the β- and α-fucose generated from dissociation of sodiated disaccharides Fuc-β-(1 → 3)-GlcNAc and Fuc-α-(1 → 4)-GlcNAc through the CID sequence 390 → 187, respectively. In this CID sequence, the disaccharide sodium ion adduct, *m*/*z* 390, is dissociated to generate the fucose sodium ion adduct, *m*/*z* 187, and then ion *m*/*z* 187 is dissociated by the subsequent CID to obtain the CID spectrum. However, the intensity ratios of ion *m*/*z* 169 to ion *m*/*z* 127 shown in Figure 4 are not the same as that shown in Figure 3. The difference of these ratios between Figure 4 and Figure 3 can be attributed to the generation of a small amount of linear fucose from disaccharides. The fucose generated from the dissociation of disaccharide can be in the ring form if a H atom is transferred from GlcNAc to the O atom of the glycosidic bond (the O1 atom of fucose). The anomericity of the fragments in the ring form is preserved during CID, as a number of studies have shown.^74−76^ Fucose in the ring form undergoes both cross-ring dissociation and dehydration in the subsequent CID. In contrast, the fucose generated from the dissociation of the disaccharide can be in the linear form if a H atom is transferred from GlcNAc to the O5 atom of fucose. The linear fucose only undergoes cross-ring dissociation, and there is no difference between α- and β-fucose. Therefore, a slightly higher branching ratio of fragment *m*/*z* 127 in the CID spectra of both the α-and β-fucose generated from disaccharides in Figure 4 compared to that in Figure 3 is expected. Similar phenomena have been found in hexose.^55^ Although the intensity ratios of ion *m*/*z* 169 to ion *m*/*z* 127 in Figure 4 are not the same at that in Figure 3, the trend that α-fucose has a larger ratio of *m*/*z* 169 to *m*/*z* 127 compared to that of β-fucose shown in Figure 4 can be used to assign the mass spectra in Figure 3. This suggests that the peaks at retention times of 6.5 and 8.0 min in the chromatogram of Figure 3a result from β-fucose and α-fucose, respectively. This assignment is further supported by the reaction mechanism obtained through quantum chemistry calculations, which provide physical insights into understanding the difference in the CID spectra between these two anomers.

**Figure 5:**
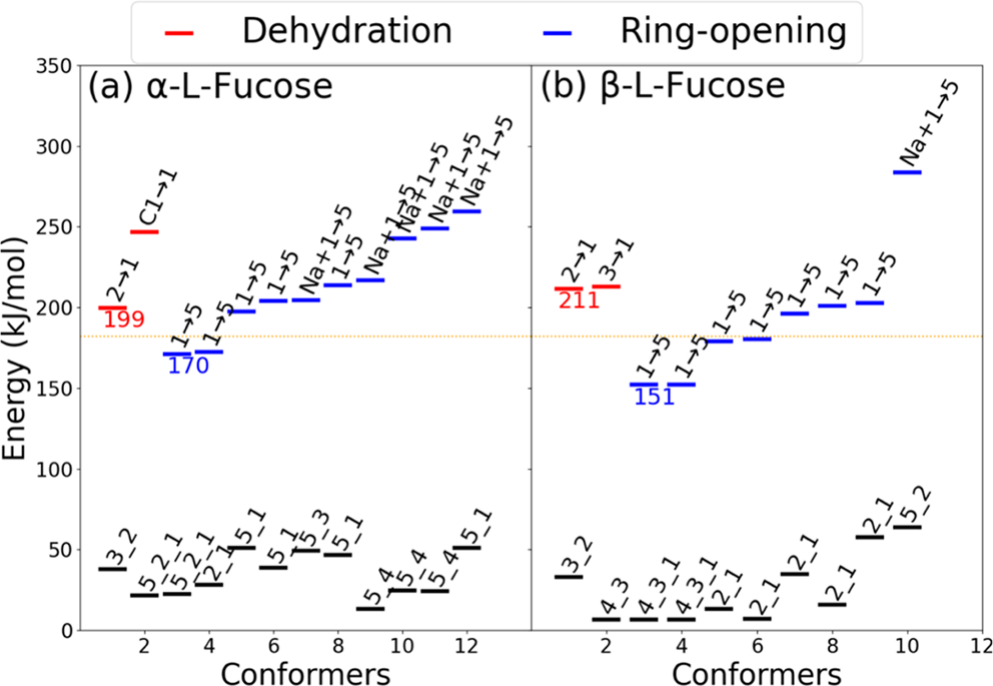
Calculated zero-point corrected energies of TSs and reactants of sodiated l-fucose using the DFT/M06-2X method. (a) α-l-fucose and (b) β-l-fucose. The energy is relative to the energy of the respective global minimum structure. The red and blue dashes represent the TS energies of different reactions. Right above the TS dashes, the H atom transfer that is responsible for the TS is illustrated, with notations X → Y or Na + X → Y indicating the H atom transfer from X(O_D_) to Y(O_A_) atoms; however, the exception in (a) with notation C1 → 1 indicates the H atom transfer from C1 to O1(O_A_) atoms. The black dashes right below each TS dash represent the energies of the reactant states leading to the TSs, where the series of numbers represent the numberings of O atoms binding to the sodium ion. The coordinate files of all conformers are provided in the Supporting Information.

**Figure 6:**
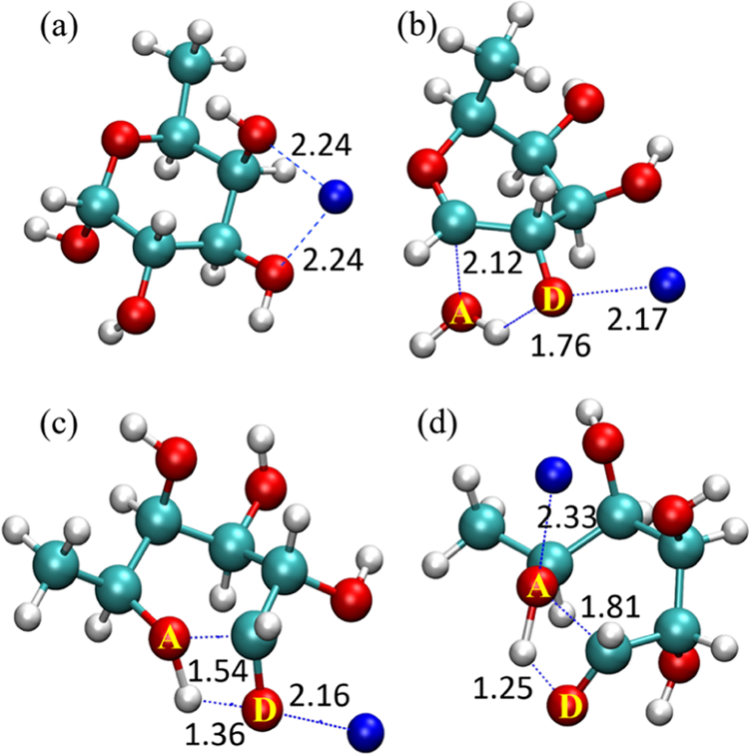
Geometries of global minimum and TS of various reactions of sodiated α-l-fucose. (a) Global minimum, (b) lowest-energy TS for dehydration, (c) lowest-energy TS for ring-opening, and (d) TS for the Na + 1 → 5-type ring-opening reaction. O atoms labeled as D and A are H atom donors and acceptors, respectively. Cyan, red, white, and blue balls represent carbon, oxygen, hydrogen, and sodium atoms, respectively.

The detailed dissociation mechanism of fucose can be understood from the calculations. Figure 5 displays the zero-point corrected energies of the TSs and their corresponding reactant states for both sodiated α- and β-l-fucose. The energies presented in Figure 5a,b are the relative energies to the global minima of sodiated α- and β-l-fucose, respectively. Figure 6a depicts the global minimum structure of sodiated α-l-fucose. The lowest-energy dehydration pathway of α-l-fucose results from the H atom transfer from O2 to O1, with TS energy 199 kJ/mol, and the TS structure is shown in Figure 6b. This TS structure indicates that the sodium ion binding to O2 weakens the O2–H bond, thereby reducing the energy barrier for the H atom transfer from O2 to O1. Following this H atom transfer, the C1–O1 bond cleaves, triggering a dehydration reaction. The second lowest TS of dehydration is presented in Figure 5a. The reaction initiates with the C1–O1 bond breaking due to the sodium ion binding to the O1, causing the nearest H atom, which is on C1, to transfer to the O1 atom. This dehydration TS, denoted as C1 → 1 in Figure 5a, is approximately 50 kJ/mol higher in energy than the lowest dehydration TS, making it a less favorable pathway in the CID. The H atom transfer from the other OH groups to O1 is unlikely to happen because they are located on the side of the ring different from that of O1; hence, the O–O distances are too large for H atom transfer.

The lowest ring-opening TS structure of sodiated α-l-fucose is shown in Figure 6c. In the reactant state leading to this TS, sodium ion binds to O1, O2, and O5 (Figure 5a). The O1 binding in the reactant state weakens the O1–H bond, promoting the transfer of the H atom to the nearest O atom, which is O5. As the geometry changes from reactant to TS, the sodium ion detaches from O5 while remaining bound to compounds O1 and O2. The energy barrier for the ring-opening reaction is only 170 kJ/mol. The other low-energy TS structures for ring-opening are similar, with the sodium ion binding to O1 and other O atoms and the H atom transferring from O1 to O5. Interestingly, we found a different type of ring-opening reaction that we did not observe in hexose in our previous studies. This new mechanism begins with the sodium ion binding to the O5 atom but not to the O1 atom. The binding to O5 weakens the O5–C1 bond, and as this bond breaks, the reaction proceeds with H atom abstraction from O1 by O5. This type of ring-opening mechanism is denoted as Na + 1 → 5 in Figure 5. The corresponding TS structure is shown in Figure 6d. Compared to the energies of TSs with the sodium ion bound to the O1 and other O atoms, the barrier for this new type of ring-opening is higher by about 40 kJ/mol.

**Figure 7:**
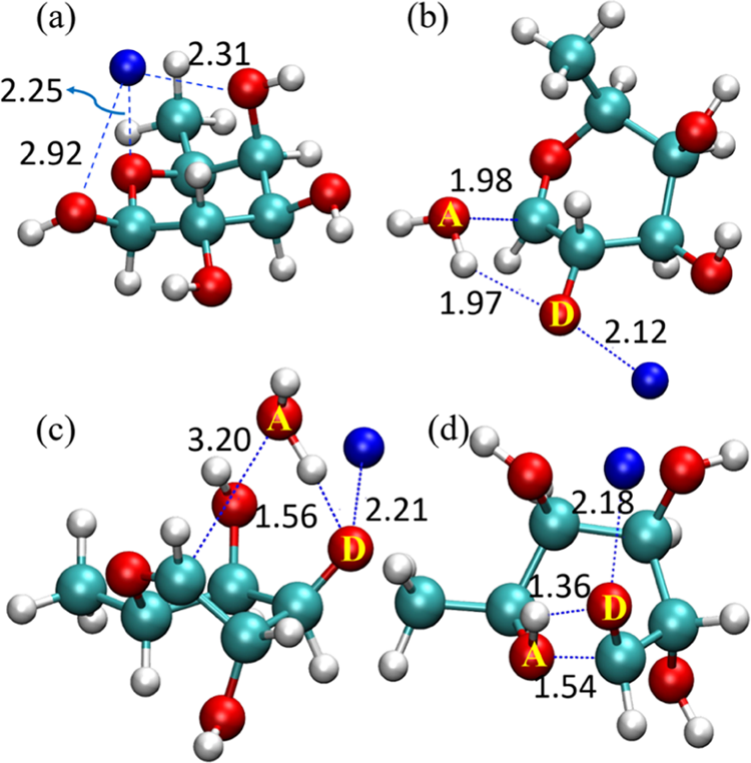
Geometries of global minimum and TS of sodiated β-l-fucose. (a) Global minimum, (b) dehydration for H atom transfer from O2 to O1, (c) dehydration for H atom transfer from O3 to O1, and (d) ring-opening reaction for H atom transfer from O1 to O5. O atoms labeled as D and A are H atom donors and acceptors, respectively. Cyan, red, white, and blue balls represent carbon, oxygen, hydrogen, and sodium atoms, respectively.

The global minimum structure of sodiated β-l-fucose is shown in Figure 7a, where the O atoms that sodium ion binds to differ from those in sodiated α-l-fucose. There are two low-barrier dehydration pathways for sodiated β-l-fucose, as shown in Figure 5b. One involves the H atom transfer from O2 to O1, while the other involves the transfer from O3 to O1. The structures of these TSs are given in Figure 7b,c, respectively. The relative energies of these two TSs are similar, around 211 kJ/mol. In the case of the H atom transfer from O2 (Figure 7b), the sodium ion binds to the O2 and O3 atoms in the reactant state, with O1 close to O2, making the H atom transfer to the O1 atom energetically favorable. On the other hand, for the H atom transfer from O3, despite O3 being separated from O1 by two C–C bonds, the reaction is still favorable because both O3 and O1 are on the same side of the plane of the fucose ring’s structure, and the sugar ring’s puckering brings O3 close to O1. In contrast, the O1 and O3 in α-l-fucose are on opposite sides, and the ring-puckering cannot bring the O3 close enough to the O1 (less than 3 Å), so we do not observe the dehydration due to the H atom transfer from the O3 to the O1 in sodiated α-l-fucose.

The difference of the lowest dehydration barrier between sodiated α-l-fucose and β-l-fucose can be attributed to the difference in the O1–C1–C2–O2 dihedral angle in the reactant states. In α-l-fucose, O1 and O2 are in cis-configuration, whereas they are in trans-configuration in β-l-fucose. The reactant in the cis-configuration has a smaller O1–O2 distance (2.8 Å) compared to the trans-configuration (3.0 Å). The longer distance of β-l-fucose results in a higher energy barrier for H atom transfer (211 kJ/mol) compared to that in α-l-fucose (199 kJ/mol). Although the ring’s puckering brings O3 close to O1, the distance is still substantial, leading to a similar barrier height of H atom transfer (212 kJ/mol).

Figure 7d shows the lowest TS for the ring-opening reaction of sodiated β-l-fucose. Similar to sodiated α-l-fucose, the lowest ring-opening TS of sodiated β-l-fucose involves H atom transfer from O1 to O5, with the sodium ion binding to O1 reducing the energy barrier for H atom transfer to O5. However, unlike in α-l-fucose, where two O atoms of OH groups bind to the sodium ion in their lowest ring-opening TS, three O atoms of OH groups are involved in that of β-l-fucose. The larger coordination number of β-l-fucose results in lower energies for both the reactant state (8 kJ/mol) and TS (151 kJ/mol) compared to α-l-fucose (20 and 170 kJ/mol, respectively).

**Figure 8:**
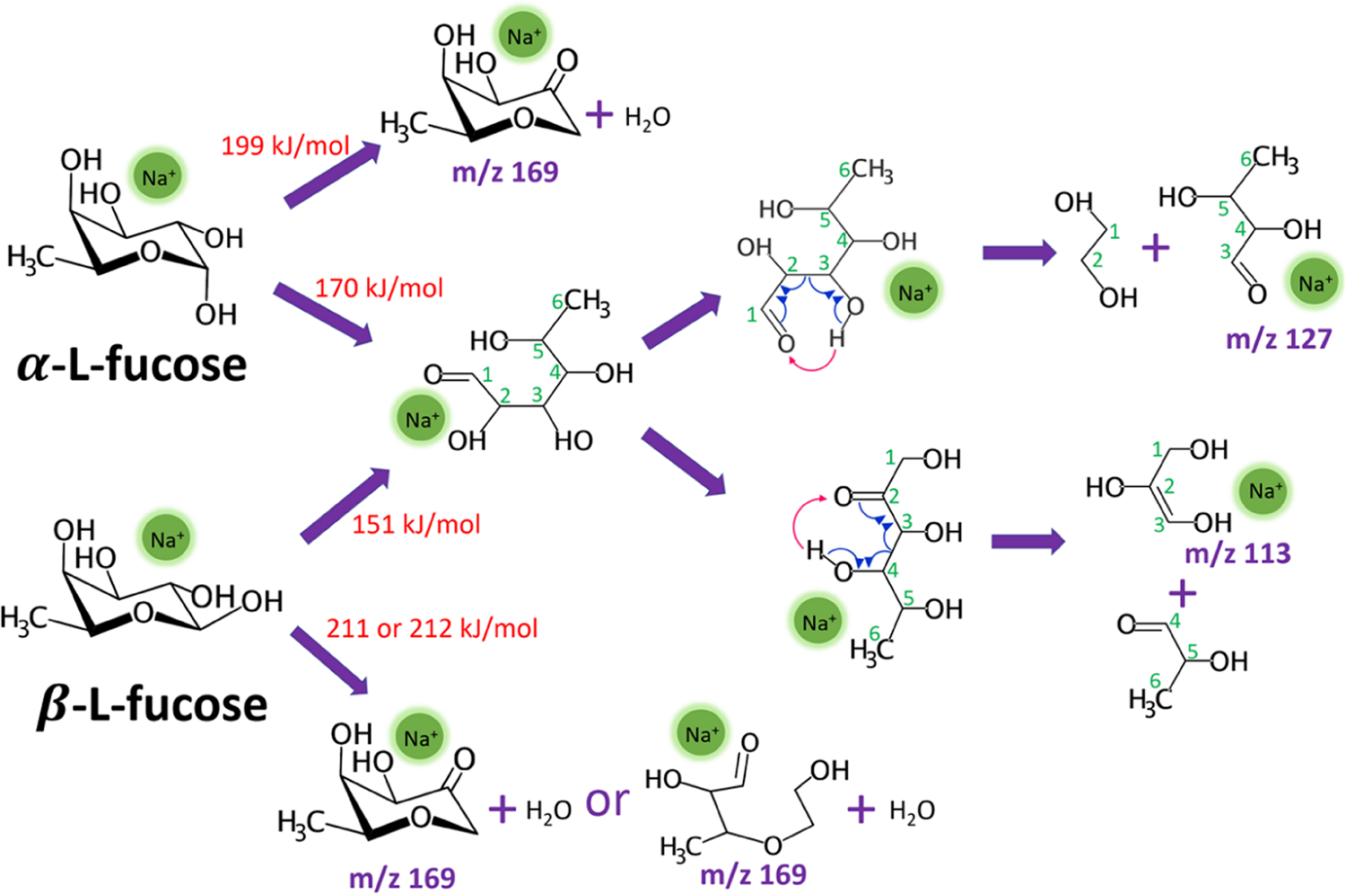
Main dissociation mechanisms of dehydration and cross-ring dissociation of α-l-fucose and β-l-fucose. Energies are calculated according to the calculations illustrated in Figure 5.

The lowest TSs depicted in Figure 5 help explain the CID mass spectrum differences between sodiated α and β-l-fucose shown in Figure 3. In CID, energy accumulates slowly in the reactants through collisions, with reactions occurring only when the energy is sufficient to overcome the dissociation barriers. Therefore, reactions predominantly follow pathways with lower barrier heights. Figure 8 summarizes the lowest dissociation pathways for the dehydration and ring-opening reactions of α- and β-l-fucose. Through Na^+^ migration around the sugar ring, different positions of Na^+^ facilitate different reactions. α-l-Fucose is more likely to undergo dehydration due to the proximity of O1 and O2 being on the same side of the sugar ring. This leads to fragment ion *m*/*z* 169. In contrast, β-l-fucose more readily undergoes ring-opening reaction because more OH groups are on the same side of the sugar ring side as O1. Our previous works^46,49,53,54^ have shown that cross-ring dissociation begins with a ring-opening reaction followed by a retro-aldol reaction. Using the same methodology as outlined in Figure 2, we identified the lowest TS of the retro-aldol reaction at 167 kJ/mol, relative to the global minimum of the sodiated linear l-fucose. The cross-ring dissociation yields fragment ions *m*/*z* 113 and 127. These differences in barrier heights suggest a higher preference for the dehydration reaction in sodiated α-l-fucose but a lower preference for ring-opening compared to that of β-l-fucose, aligning with experimental results. The correlation of cis- and trans-configurations of O1 and O2 to the relative branching ratios of dehydration and cross-ring dissociation in CID have been observed in aldohexose and ketohexose in the pyranose form, but not in ketohexose in the furanose form and *N*-acetylhexosamine.^53,77,78^ This study demonstrates that the correlation extends to deoxyhexoses for anomericity determination.

## Conclusions

Through HPLC and tandem mass spectrometry, we investigated the CID spectra of α- and β-l-fucose sodium adducts. In the monosaccharide experiment, we found that the major difference in the mass spectra pattern between these two molecules is the relative intensity of fragment ions *m*/*z* 169 and *m*/*z* 127, corresponding to dehydration and cross-ring dissociation, respectively. We can ascertain that the ratio of dehydration to cross-ring cleavage is significantly higher in the case of α-l-fucose compared to that of β-l-fucose through the disaccharide experiments. To explain this difference found in mass spectra, we conducted quantum chemistry calculations of α-l-fucose and β-l-fucose sodium ion adducts, focusing on the dehydration and ring-opening reactions (the first step of cross-ring dissociation that involves multiple steps). The calculation results suggest that the dehydration barrier depends on the O1–O2 distance, while the barrier of the ring-opening reaction depends on the number of OH groups on the same ring side of O1. In the case of α-l-fucose, O1 and O2 are in the cis-configuration, the distance of which is short, but only one atom, i.e., O2, is on the same side of O1. In contrast, O1 and O2 are in the trans-configuration in β-l-fucose, whose distance is large, but there are two O atoms (i.e., O3 and O4) on the same side of O1 in β-l-fucose. Thus, the dehydration barrier of α-fucose is lower than that of β-fucose, while the ring-opening barrier of α-fucose is higher than that of β-fucose. The barrier of dehydration is higher than that of the ring-opening barrier by 29 kJ/mol for α-fucose, but it becomes 60 kJ/mol for β-fucose. This qualitatively explains the relative intensity differences between the fragments *m*/*z* 169 and *m*/*z* 127 in the CID spectra of these two molecules, and the change in intensity ratios of fragments *m*/*z* 169 and *m*/*z* 127 provides a simple method to determine the anomericity of fucose.

## Supporting Information Available

The coordinate files of all conformers, including reactants and transition states. (ZIP)
The coordinate data. (PDF)
