# Supplementary material for: Collision-Induced Dissociation of Fucose and Identification of Anomericity
Source: J Phys Chem A. 2024 May 1;128(19):3812–20. doi: 10.1021/acs.jpca.4c00640 (PMC11103703; doi:10.1021/acs.jpca.4c00640)
Supplement: Supplementary file 1 — jp4c00640_si_001.zip [file jp4c00640_si_001.zip › Supporting Information.docx]

Supporting Information

**Collision induced dissociation of fucose and identification of anomericity**

Hock Seng Nguan^1^, Jien-Lian Chen^1^, and Chi-Kung Ni*^1,2^

1 Institute of Atomic and Molecular Sciences, Academia Sinica, P. O. Box 23-166, Taipei 10617, Taiwan. E-mail: ckni@po.iams.sinica.edu.tw

2 Department of Chemistry, National Tsing Hua University, Hsinchu 30013, Taiwan
