# Supplementary material for: Collision-Induced Dissociation of Fucose and Identification of Anomericity
Source: J Phys Chem A. 2024 May 1;128(19):3812–20. doi: 10.1021/acs.jpca.4c00640 (PMC11103703; doi:10.1021/acs.jpca.4c00640)
Supplement: Supplementary file 2 — jp4c00640_si_002.pdf [file jp4c00640_si_002.pdf]

Supporting Information

Collision induced dissociation of fucose and identification of anomericity

Hock Seng Nguan[1], Jien-Lian Chen[1], and Chi-Kung Ni*[1,2]

1 Institute of Atomic and Molecular Sciences, Academia Sinica, P. O. Box 23-166, Taipei 10617, Taiwan. E-mail: ckni@po.iams.sinica.edu.tw

2 Department of Chemistry, National Tsing Hua University, Hsinchu 30013, Taiwan

This supporting information contains the xyz coordinates for the global minima, reactants and transition states for sodiated α- and β-L-fucose.

alpha_L_fucose/globalminimum.xyz

```
24
eng=   -773.854775
C   1.655952   -0.656274   -0.638122
O   1.53739   0.745443   -0.737199
C   0.971871   1.376785   0.415816
C   1.091594   2.874253   0.22348
C   -0.468334   0.886608   0.565341
O   -1.269895   1.254317   -0.569951
C   -0.460532   -0.627724   0.705692
O   -1.815372   -1.094065   0.728693
C   0.276117   -1.28959   -0.455595
O   0.328869   -2.678975   -0.259033
O   2.412499   -1.060178   0.467066
H   1.523746   1.066297   1.308618
H   -0.931793   1.347571   1.442173
H   0.031238   -0.889967   1.648575
H   -0.294702   -1.123829   -1.373903
H   2.110935   -0.978367   -1.576624
H   3.350501   -0.916478   0.30477
H   1.085362   -2.872897   0.309956
H   -1.785963   -2.056736   0.808646
H   -0.683664   1.427226   -1.318445
H   0.569656   3.206999   -0.675638
H   0.673259   3.39988   1.083382
H   2.141828   3.145551   0.124356
Na   -3.24894   0.229824   -0.36565
```

alpha_L_fucose/1.dehyd_reactant.xyz

24
eng= -773.840322
C 0.358061 1.769938 0.234441
O 1.638543 1.297278 0.509028
C 1.964348 -0.088102 0.284384
C 1.904325 -0.892221 1.575448
C 1.1041 -0.667088 -0.846305
O 1.273516 -2.06578 -0.972538
C -0.406977 -0.434916 -0.621631
O -1.0937 -1.595723 -0.14422
C -0.677302 0.67255 0.388946
O -2.019591 1.137458 0.231367
O 0.198049 2.209603 -1.096081
H 3.001371 -0.063117 -0.058674
H 1.365744 -0.15347 -1.774667
H -0.848678 -0.141292 -1.578574
H -0.609654 0.274559 1.402094
H 0.180293 2.591245 0.933387
H 0.762235 2.973711 -1.256179
H -2.021528 1.815908 -0.458382
H -0.570393 -2.372644 -0.395898
H 2.026314 -2.27676 -1.530872
H 2.402337 -1.852009 1.436523
H 2.420994 -0.342167 2.362137
H 0.883231 -1.099054 1.901724
Na -3.131467 -0.852596 0.427748

alpha_L_fucose/1.dehyd_ts.xyz

24
eng= -773.778662
C -0.089401 1.651561 -0.558535
O -1.353275 1.593802 -0.457313
C -2.027244 0.309076 -0.165542
C -2.277075 -0.451449 -1.45176
C -1.210729 -0.443049 0.891286
O -1.729202 -1.744328 1.064724
C 0.281547 -0.564247 0.525986
O 0.660446 -1.848685 0.039745
C 0.780344 0.431854 -0.544855
O 2.099539 0.70189 -0.396745
O 0.830405 2.1646 1.286498
H -2.972522 0.64238 0.261003
H -1.259014 0.132109 1.820044
H 0.857031 -0.347528 1.429479
H 0.52469 -0.018282 -1.530655
H 0.290367 2.589673 -0.953555
H 0.957334 3.080523 1.55282
H 1.676559 1.816551 0.894569
H 0.05791 -2.508384 0.410941
H -2.335244 -1.781674 1.809506
H -2.962528 -1.270087 -1.232499
H -2.742916 0.201798 -2.189843
H -1.368469 -0.886671 -1.867619
Na 2.951713 -1.290101 -0.44354

alpha_L_fucose/2.dehyd_reactant.xyz

24
eng= -773.846591
C 0.620095 -0.337935 0.866378
O 0.469257 -1.139727 -0.29897
C -0.910113 -1.228192 -0.75283
C -1.554049 -2.491106 -0.218318
C -1.68987 0.047488 -0.394005
O -2.208445 -0.063431 0.914133
C -0.771038 1.265149 -0.489107
O -1.442633 2.445398 -0.096349
C 0.455736 1.119517 0.435767
O 1.649663 1.46085 -0.278993
O 1.929238 -0.54506 1.318691
H -0.833705 -1.270612 -1.84154
H -2.507624 0.14804 -1.118961
H -0.396722 1.345394 -1.516721
H 0.338426 1.760939 1.311471
H -0.118146 -0.609155 1.621317
H 1.940687 -1.264018 1.95839
H 1.762172 2.415542 -0.291728
H -1.9974 2.770791 -0.811985
H -2.652705 0.763057 1.136621
H -1.589788 -2.477651 0.871264
H -2.581085 -2.567273 -0.580745
H -0.996016 -3.364322 -0.555323
Na 2.686427 -0.4695 -0.84886

alpha_L_fucose/2.dehyd_ts.xyz

24
eng= -773.760899
C 0.438052 -0.912886 0.481181
O -0.582287 -1.558751 0.125938
C -1.790186 -0.992308 -0.545358
C -2.97241 -1.74628 0.010759
C -1.870843 0.518486 -0.395096
O -2.350777 0.814531 0.89075
C -0.489848 1.146158 -0.604412
O -0.509154 2.539595 -0.377321
C 0.426063 0.578309 0.468577
O 1.739432 1.098809 0.390242
O 2.638688 -1.676823 -0.246817
H -1.610913 -1.245732 -1.592489
H -2.560571 0.875358 -1.171764
H -0.094326 0.904413 -1.59807
H -0.020864 0.834746 1.444512
H 1.426732 -1.496 0.618193
H 2.735072 -2.558405 -0.611069
H 1.641572 2.057857 0.460718
H -0.744788 3.016838 -1.17908
H -2.458783 1.768513 0.97722
H -3.089884 -1.540592 1.07347
H -3.875583 -1.416923 -0.506259
H -2.845194 -2.815666 -0.151768
Na 3.778667 0.029803 -0.047259

alpha_L_fucose/1.ringopen_reactant.xyz

24
eng=   -773.851191
C   0.508886   -1.002253   0.746521
O   -0.808474   -1.388523   0.637623
C   -1.571519   -0.86109   -0.463335
C   -2.979659   -1.387362   -0.28658
C   -1.502691   0.667802   -0.511953
O   -2.063449   1.296818   0.623795
C   -0.045921   1.108   -0.525477
O   0.1151   2.505378   -0.499582
C   0.668023   0.510292   0.680442
O   2.060592   0.83353   0.619035
O   1.316632   -1.566809   -0.30795
H   -1.14192   -1.235196   -1.401647
H   -1.992102   1.009829   -1.431801
H   0.428188   0.747616   -1.443824
H   0.232187   0.921438   1.595191
H   0.860639   -1.394038   1.7034
H   1.055476   -2.49127   -0.401809
H   2.115503   1.798877   0.609881
H   -0.585957   2.870204   0.057215
H   -3.023381   1.253124   0.601446
H   -3.625858   -1.020358   -1.086655
H   -2.982987   -2.476489   -0.313135
H   -3.379341   -1.073767   0.679468
Na   3.328512   -0.59686   -0.508073

alpha_L_fucose/1.ringopen_ts.xyz

```
24
eng=   -773.789646
C   0.659324   -0.917135   0.725323
O   -0.599094   -1.540783   0.100995
C   -1.698815   -0.848319   -0.59086
C   -2.995575   -1.454431   -0.104361
C   -1.609317   0.664006   -0.399726
O   -2.017519   1.099421   0.882436
C   -0.171903   1.136872   -0.551046
O   -0.061061   2.539904   -0.575827
C   0.672042   0.608101   0.605079
O   2.034543   0.989468   0.406116
O   1.511013   -1.576588   -0.08915
H   -1.543259   -1.056046   -1.652734
H   -2.219837   1.127541   -1.18308
H   0.238251   0.771871   -1.498163
H   0.30345   1.046324   1.535734
H   0.635392   -1.263031   1.761534
H   0.272423   -1.994051   -0.470992
H   2.038504   1.950943   0.305962
H   -0.650162   2.900302   0.099643
H   -2.976397   1.105899   0.954385
H   -3.839115   -0.986689   -0.618593
H   -3.019859   -2.522921   -0.315088
H   -3.102354   -1.310752   0.971601
Na   3.435034   -0.635939   -0.344838
```

alpha_L_fucose/2.ringopen_reactant.xyz

24
eng= -773.848307
C 0.505205 -0.987353 0.717263
O -0.801804 -1.391234 0.612698
C -1.58834 -0.85197 -0.470325
C -2.988022 -1.396138 -0.290695
C -1.537387 0.674326 -0.467689
O -2.149273 1.142155 0.706596
C -0.076862 1.121238 -0.529798
O 0.073156 2.523619 -0.390437
C 0.665765 0.527381 0.652332
O 2.061452 0.839037 0.577341
O 1.318698 -1.548779 -0.339631
H -1.158077 -1.198857 -1.418653
H -2.067321 1.029409 -1.363116
H 0.377102 0.769016 -1.465104
H 0.244065 0.938948 1.572425
H 0.868413 -1.371258 1.673311
H 1.028484 -2.461704 -0.459015
H 2.138281 1.798068 0.657902
H -0.138523 2.970554 -1.215611
H -2.101161 2.103802 0.724636
H -3.396859 -1.050663 0.657976
H -3.63019 -1.047499 -1.101309
H -2.970566 -2.485639 -0.301161
Na 3.355124 -0.639768 -0.451032

alpha_L_fucose/2.ringopen_ts.xyz

```
24
eng=   -773.789114
C   0.643986   -0.898936   0.714095
O   -0.610057   -1.523647   0.114033
C   -1.703571   -0.828456   -0.594551
C   -3.004447   -1.458876   -0.1567
C   -1.625754   0.67033   -0.349026
O   -2.072361   0.938236   0.957144
C   -0.191696   1.159315   -0.54564
O   -0.091645   2.56703   -0.429788
C   0.681544   0.627091   0.578605
O   2.048176   0.981151   0.353854
O   1.494152   -1.576062   -0.093657
H   -1.513306   -1.006262   -1.656281
H   -2.278897   1.14415   -1.094488
H   0.197372   0.811856   -1.511951
H   0.341695   1.078589   1.512354
H   0.630753   -1.2231   1.757374
H   0.254183   -1.998654   -0.455428
H   2.091375   1.945361   0.369326
H   -0.365959   2.998138   -1.244823
H   -2.105558   1.891658   1.091561
H   -3.157328   -1.300272   0.909573
H   -3.831414   -0.997898   -0.700935
H   -2.997769   -2.526855   -0.372658
Na   3.435305   -0.684835   -0.336092
```

alpha_L_fucose/3.ringopen_reactant.xyz

24
eng= -773.834973
C -0.684458 -0.100761 1.128758
O 0.277292 0.887584 1.017045
C 0.728788 1.151353 -0.320975
C 1.520156 2.440358 -0.297805
C 1.536633 -0.06449 -0.785626
O 2.84737 -0.067498 -0.274709
C 0.820537 -1.398521 -0.379876
O 1.402819 -1.98255 0.755066
C -0.632646 -1.17615 0.031949
O -1.437738 -0.780234 -1.093397
O -1.996319 0.481165 1.111546
H -0.141165 1.266016 -0.975979
H 1.641857 -0.021831 -1.87178
H 0.844546 -2.099638 -1.226177
H -1.030139 -2.106025 0.44495
H -0.522563 -0.582885 2.093125
H -2.048812 1.075185 1.869318
H -1.247054 -1.378138 -1.824724
H 2.352095 -2.060002 0.604488
H 2.813541 0.159463 0.66253
H 2.331995 2.387463 0.427392
H 1.955278 2.629657 -1.28034
H 0.871039 3.273365 -0.028386
Na -3.300188 0.367173 -0.66158

alpha_L_fucose/3.ringopen_ts.xyz

```
24
eng=   -773.77962
C   -0.819335   -0.210027   1.087667
O   0.193483   0.93062   0.982318
C   0.763741   1.154496   -0.349752
C   1.579079   2.425049   -0.296787
C   1.559805   -0.089513   -0.747701
O   2.859352   -0.101322   -0.223099
C   0.818578   -1.412604   -0.371663
O   1.326384   -1.950647   0.827495
C   -0.667238   -1.208965   -0.058611
O   -1.425866   -0.748055   -1.181997
O   -1.907708   0.595575   1.079852
H   -0.080219   1.259748   -1.037379
H   1.683192   -0.043102   -1.83215
H   0.92234   -2.132597   -1.19189
H   -1.070872   -2.163164   0.292587
H   -0.577323   -0.683606   2.040143
H   -0.839609   1.424756   1.101431
H   -1.295063   -1.357799   -1.916509
H   2.197174   -2.328464   0.666916
H   2.807424   -0.108553   0.740185
H   2.369567   2.348865   0.448348
H   2.045119   2.594566   -1.26899
H   0.94423   3.279785   -0.060596
Na   -3.352628   0.377234   -0.494326
```

alpha_L_fucose/4.ringopen_reactant.xyz

24
eng= -773.839239
C 0.540006 -0.793095 -0.579573
O 1.001178 0.401265 0.05245
C 0.04349 1.20948 0.778426
C 0.540153 2.637924 0.738164
C -1.330471 1.039931 0.148576
O -1.205956 1.380723 -1.210592
C -1.803939 -0.415624 0.303985
O -2.770515 -0.616705 -0.704557
C -0.631201 -1.413794 0.166721
O -0.081235 -1.796665 1.41832
O 1.65614 -1.650713 -0.588576
H 0.007642 0.831915 1.80635
H -2.039244 1.702561 0.658694
H -2.231267 -0.540514 1.305253
H -0.968263 -2.286002 -0.403375
H 0.271571 -0.57366 -1.613047
H 1.602406 -2.187071 0.217672
H -0.664238 -2.413204 1.87075
H -3.414287 -1.281022 -0.446314
H -2.038225 1.167429 -1.649647
H 0.549404 3.001462 -0.289726
H -0.116265 3.277417 1.330527
H 1.542236 2.708177 1.16666
Na 3.141219 0.114578 -0.457086

alpha_L_fucose/4.ringopen_ts.xyz

24
eng= -773.777115
C 0.845385 -0.177038 -0.411804
O 0.267604 1.137477 -0.037833
C -1.055615 1.225613 0.600378
C -1.582341 2.617382 0.34371
C -1.888703 0.12284 -0.040918
O -1.847532 0.34013 -1.429987
C -1.331618 -1.271058 0.306587
O -1.768844 -2.132317 -0.724821
C 0.204491 -1.270331 0.433322
O 0.498831 -1.047073 1.792286
O 2.120732 0.169114 9.5e-05
H -0.920667 1.016626 1.663587
H -2.912802 0.204241 0.341911
H -1.719048 -1.578663 1.282143
H 0.58948 -2.237916 0.087903
H 0.708555 -0.316643 -1.484722
H 1.353244 1.178022 0.386653
H 1.446601 -0.893397 1.872363
H -1.903315 -3.022078 -0.389308
H -2.258976 -0.41999 -1.858488
H -1.709333 2.777954 -0.726238
H -2.547837 2.732729 0.839622
H -0.901674 3.366842 0.749055
Na 4.132713 0.179015 -0.631823

alpha_L_fucose/5.ringopen_reactant.xyz

24
eng= -773.85183
C -1.338155 0.129295 0.941367
O -0.101541 0.839383 0.85307
C 0.219831 1.147741 -0.521715
C 1.207496 2.293445 -0.544982
C 0.731254 -0.16008 -1.149496
O 2.106169 -0.37896 -0.811339
C -0.061441 -1.375369 -0.586556
O 0.583401 -1.887601 0.588292
C -1.462077 -0.93963 -0.174988
O -2.073645 -0.452698 -1.338973
O -2.422112 0.979749 0.790116
H -0.69723 1.448066 -1.029596
H 0.606234 -0.12458 -2.233116
H -0.122181 -2.153778 -1.346978
H -2.008472 -1.795958 0.233997
H -1.322153 -0.344486 1.924981
H -2.516578 1.552645 1.557463
H -2.890528 0.000454 -1.097923
H 0.786317 -2.818086 0.465802
H 2.675228 0.008504 -1.482197
H 2.118811 2.070127 0.015907
H 1.494812 2.523033 -1.573748
H 0.749749 3.182913 -0.113732
Na 1.873306 -0.265478 1.477093

alpha_L_fucose/5.ringopen_ts.xyz

24
eng= -773.7769
C -1.53245 -0.012669 0.967664
O 0.044433 0.879396 0.926262
C 0.371963 1.188793 -0.447889
C 1.477191 2.225308 -0.498436
C 0.717291 -0.133343 -1.142949
O 2.040362 -0.55653 -0.791597
C -0.23138 -1.290885 -0.727937
O 0.296826 -1.966235 0.420965
C -1.627409 -0.786717 -0.336264
O -2.133609 -0.02925 -1.391132
O -2.159767 1.10365 1.112139
H -0.524757 1.590261 -0.924761
H 0.638655 0.009859 -2.2225
H -0.319565 -1.98891 -1.560852
H -2.257647 -1.662258 -0.120978
H -1.317702 -0.629674 1.84597
H -0.975207 1.502606 1.212868
H -2.836544 0.544536 -1.057546
H 0.461355 -2.888618 0.208639
H 2.676905 -0.200442 -1.418165
H 2.382613 1.885796 0.009658
H 1.7298 2.455786 -1.536611
H 1.144345 3.14799 -0.023154
Na 1.767323 -0.578008 1.499019

alpha_L_fucose/6.ringopen_reactant.xyz

24
eng= -773.840872
C -0.975039 -0.342069 0.678723
O -0.840699 0.760864 -0.220415
C 0.495677 1.297168 -0.369444
C 0.952918 2.045172 0.873055
C 1.409767 0.161676 -0.852992
O 2.73403 0.659282 -0.843627
C 1.331846 -1.0844 0.033817
O 1.859732 -0.841837 1.313924
C -0.112837 -1.52541 0.255079
O -0.71719 -2.026982 -0.924739
O -2.338844 -0.67401 0.62156
H 0.405164 2.009143 -1.191922
H 1.089695 -0.11616 -1.862516
H 1.874492 -1.901545 -0.463189
H -0.133408 -2.265815 1.061836
H -0.729884 -0.02756 1.694271
H -2.412752 -1.45442 0.049824
H -0.393012 -2.912614 -1.113624
H 2.72321 -0.423721 1.208234
H 3.232215 0.309148 -1.586779
H 1.004866 1.407598 1.754129
H 1.948379 2.452065 0.692822
H 0.275198 2.878476 1.068267
Na -3.006763 1.246733 -0.417673

alpha_L_fucose/6.ringopen_ts.xyz

24
eng= -773.77339
C -1.013356 0.053319 0.479196
O -0.344325 1.064344 -0.37964
C 1.097956 1.155493 -0.656553
C 1.694936 2.121962 0.347091
C 1.707957 -0.25959 -0.681962
O 3.099399 -0.094716 -0.477092
C 1.146279 -1.187042 0.400153
O 1.402575 -0.696938 1.695294
C -0.365668 -1.310347 0.269962
O -0.624739 -1.788843 -1.024618
O -2.222328 0.195895 -0.168133
H 1.143077 1.579582 -1.659903
H 1.50059 -0.714418 -1.65333
H 1.588235 -2.180348 0.254829
H -0.744134 -1.98919 1.044096
H -0.976793 0.39423 1.514543
H -1.359881 0.913582 -0.909796
H -1.567761 -1.697942 -1.19502
H 2.354684 -0.574127 1.786509
H 3.585105 -0.75525 -0.978126
H 1.542008 1.771929 1.367486
H 2.764778 2.215839 0.16566
H 1.228749 3.100215 0.227493
Na -4.286539 0.459562 0.174705

alpha_L_fucose/7.ringopen_reactant.xyz

```
24
eng=   -773.851674
C   -1.335871   -0.162611   -0.940285
O   -0.097145   -0.859569   -0.836322
C   0.232622   -1.131979   0.544844
C   1.228444   -2.269496   0.595255
C   0.731514   0.189977   1.14604
O   2.114692   0.312618   0.812919
C   -0.073747   1.391439   0.559577
O   0.612095   1.881335   -0.601226
C   -1.469906   0.93528   0.147018
O   -2.086782   0.4813   1.320367
O   -2.415799   -1.014359   -0.765665
H   -0.681832   -1.424232   1.061404
H   0.601512   0.16226   2.229276
H   -0.148824   2.18383   1.306931
H   -2.024383   1.775294   -0.288817
H   -1.325759   0.284272   -1.937047
H   -2.492029   -1.625215   -1.505426
H   -2.891883   0.003182   1.087231
H   0.366177   2.796852   -0.760649
H   2.540384   0.973484   1.367048
H   2.134707   -2.056166   0.024813
H   1.533155   -2.451562   1.627115
H   0.767875   -3.174234   0.200189
Na   1.884188   0.220544   -1.469758
```

alpha_L_fucose/7.ringopen_ts.xyz

24
eng=   -773.772241
C   -0.987161   -0.949605   -0.935731
O   0.587856   -1.099842   -0.0638
C   0.771161   -0.291019   1.118119
C   2.194478   -0.424784   1.630925
C   0.416838   1.127033   0.672895
O   1.208286   1.403024   -0.488318
C   -1.084759   1.281447   0.341233
O   -1.154003   2.238092   -0.699198
C   -1.800361   -0.027196   -0.054537
O   -2.164846   -0.675734   1.12951
O   -1.147334   -2.22298   -0.812768
H   0.06118   -0.614043   1.881556
H   0.685139   1.835479   1.461328
H   -1.59639   1.629965   1.242548
H   -2.680783   0.249141   -0.656338
H   -0.679495   -0.523742   -1.900327
H   -0.095873   -2.092432   -0.122459
H   -2.461645   -1.570103   0.914059
H   -1.972618   2.740006   -0.652513
H   0.827996   2.190156   -0.904145
H   2.926544   0.035745   0.959215
H   2.290853   0.084187   2.59158
H   2.4474   -1.475051   1.780316
Na   2.231531   -0.356823   -1.432332

alpha_L_fucose/8.ringopen_reactant.xyz

24
eng= -773.849707
C 0.652446 -1.381963 0.453015
O -0.753546 -1.154455 0.303734
C -1.084128 -0.451743 -0.919687
C -2.590703 -0.474254 -1.087265
C -0.485387 0.945107 -0.836077
O -1.061187 1.602419 0.302646
C 1.025072 0.860026 -0.65324
O 1.461406 2.178113 -0.416571
C 1.38604 -0.042321 0.530541
O 2.77286 -0.210472 0.636091
O 1.181588 -2.074159 -0.627826
H -0.617244 -0.980854 -1.753348
H -0.721212 1.513061 -1.740879
H 1.467574 0.449734 -1.570225
H 1.076404 0.468237 1.447905
H 0.758842 -1.943467 1.384275
H 0.999363 -3.01693 -0.559671
H 3.060671 -0.827887 -0.048167
H 2.403163 2.172554 -0.209547
H -0.595235 2.444201 0.404356
H -2.854826 -0.07958 -2.069307
H -2.96309 -1.496185 -1.017338
H -3.111458 0.154692 -0.357948
Na -1.921086 0.152885 1.780144

alpha_L_fucose/8.ringopen_ts.xyz

24
eng= -773.762362
C 0.812263 -1.478837 0.410459
O -0.959741 -1.162653 -0.008691
C -1.145628 -0.136584 -1.01617
C -2.630298 0.08299 -1.254461
C -0.406013 1.111569 -0.566479
O -0.875542 1.446567 0.74808
C 1.100259 0.899993 -0.544211
O 1.647555 2.143684 -0.175052
C 1.556245 -0.157913 0.468536
O 2.931126 -0.420943 0.323191
O 0.896449 -2.242169 -0.619776
H -0.680674 -0.48261 -1.945305
H -0.629032 1.933487 -1.253776
H 1.424424 0.602317 -1.550511
H 1.4375 0.268238 1.467267
H 0.595293 -1.940779 1.382896
H -0.337918 -2.068435 -0.575121
H 3.071196 -0.967985 -0.461302
H 2.605774 2.066131 -0.096584
H -0.41196 2.246947 1.028004
H -2.775948 0.724327 -2.125338
H -3.129518 -0.867404 -1.444746
H -3.117679 0.587663 -0.413363
Na -2.081017 -0.195009 1.714529

alpha_L_fucose/9.ringopen_reactant.xyz

24
eng= -773.84563
C -0.646382 1.418224 0.433542
O 0.765036 1.141895 0.335897
C 1.104597 0.453039 -0.892632
C 2.614303 0.441519 -1.035691
C 0.469671 -0.929073 -0.84623
O 1.028675 -1.632345 0.274195
C -1.038649 -0.815899 -0.664988
O -1.498915 -2.129174 -0.445111
C -1.387297 0.077126 0.524476
O -2.783111 0.200219 0.513946
O -1.106194 2.10893 -0.665763
H 0.666869 1.006668 -1.72558
H 0.691186 -1.478745 -1.765575
H -1.481184 -0.377536 -1.566912
H -1.047032 -0.429819 1.439026
H -0.751004 1.996109 1.357215
H -0.902303 3.046468 -0.593476
H -3.111916 0.563184 1.34106
H -2.456866 -2.115659 -0.340408
H 0.540565 -2.463644 0.354627
H 2.883445 0.062447 -2.022577
H 3.009668 1.45255 -0.938323
H 3.11161 -0.216417 -0.315485
Na 1.900508 -0.221039 1.778203

alpha_L_fucose/9.ringopen_ts.xyz

```
24
eng=   -773.760062
C   -0.838193   1.51758   0.390456
O   0.99668   1.15791   0.034469
C   1.160686   0.153419   -0.989889
C   2.639376   -0.102526   -1.237781
C   0.392686   -1.089318   -0.571317
O   0.852966   -1.468677   0.735123
C   -1.110507   -0.860688   -0.552095
O   -1.671962   -2.103171   -0.198633
C   -1.574744   0.18781   0.464082
O   -2.94321   0.378322   0.189889
O   -0.834249   2.263356   -0.645176
H   0.705837   0.524025   -1.915105
H   0.599976   -1.898548   -1.278212
H   -1.436328   -0.538278   -1.548504
H   -1.425075   -0.228603   1.467253
H   -0.617667   1.980657   1.363681
H   0.367398   2.096039   -0.539852
H   -3.400514   0.78105   0.934486
H   -2.633213   -2.04044   -0.233652
H   0.379776   -2.271258   0.990404
H   2.76759   -0.725761   -2.124426
H   3.163337   0.83889   -1.405084
H   3.115784   -0.640938   -0.410941
Na   2.108679   0.133053   1.704894
```

alpha_L_fucose/10.ringopen_reactant.xyz

24
eng= -773.835307
C 0.837693 -0.87425 -0.574977
O 0.663313 0.5307 -0.533878
C -0.066514 1.017435 0.624367
C 0.055889 2.523681 0.636832
C -1.522341 0.528453 0.55203
O -2.355488 1.397823 -0.160209
C -1.594186 -0.899687 -0.058091
O -1.771923 -0.80986 -1.463444
C -0.27981 -1.65216 0.139516
O 0.087467 -1.753751 1.505885
O 2.103122 -1.170743 -0.016455
H 0.395513 0.589007 1.51982
H -1.903883 0.501644 1.5752
H -2.415486 -1.45328 0.402858
H -0.343524 -2.640829 -0.322065
H 0.859498 -1.139082 -1.630754
H 1.960319 -1.557211 0.862132
H -0.347799 -2.505343 1.918854
H -2.645422 -1.122648 -1.711192
H -2.221655 1.229567 -1.101136
H -0.303567 2.945904 -0.300998
H -0.549698 2.939224 1.443248
H 1.091681 2.834255 0.806483
Na 2.911609 0.905874 -0.5487

alpha_L_fucose/10.ringopen_ts.xyz

24
eng= -773.75596
C 1.228554 1.304536 -0.752514
O -1.204924 0.468295 -0.039617
C -0.977819 -0.715547 0.658845
C -2.201213 -1.636906 0.654283
C 0.216954 -1.423984 -0.032835
O 0.13118 -1.183257 -1.426512
C 1.580087 -0.913634 0.425863
O 2.597686 -1.335928 -0.439088
C 1.680601 0.643996 0.484023
O 1.041329 1.278784 1.555608
O 0.539834 2.352655 -0.70875
H -0.698209 -0.518667 1.704004
H 0.199162 -2.505322 0.134684
H 1.799858 -1.262806 1.439606
H 2.769661 0.829465 0.473146
H 1.588644 0.992297 -1.729514
H 0.187129 2.433643 0.211676
H 1.634113 1.359165 2.308964
H 2.192478 -1.621117 -1.268313
H -0.5343 -0.467944 -1.480067
H -2.47616 -1.903302 -0.372829
H -2.023291 -2.565273 1.201206
H -3.050519 -1.149443 1.149762
Na -3.235671 0.925831 -0.357404

beta_L_fucose/globalminimum.xyz

```
24
eng=   -773.854153
C   -0.52943   1.270742   -0.631668
O   0.861222   0.984899   -0.488778
C   1.263925   -0.282605   -1.037444
C   2.774408   -0.35722   -0.974018
C   0.554763   -1.368904   -0.239354
O   0.906404   -1.184812   1.140463
C   -0.951964   -1.228615   -0.39718
O   -1.534692   -2.184723   0.454466
C   -1.394379   0.176159   -0.003902
O   -2.737721   0.306403   -0.384744
O   -0.762981   2.424851   0.112377
H   0.929266   -0.33711   -2.08043
H   0.874528   -2.359708   -0.573216
H   -1.223824   -1.408937   -1.44533
H   -1.29425   0.263203   1.088377
H   -0.77056   1.400519   -1.693362
H   -0.55358   3.210664   -0.403977
H   -3.123353   1.089579   0.021111
H   -2.493923   -2.149547   0.368837
H   0.454456   -1.876503   1.641729
H   3.127901   -0.368295   0.059783
H   3.120546   -1.276378   -1.448587
H   3.215397   0.490653   -1.49735
Na   1.234146   1.026507   1.726686
```

beta_L_fucose/1.dehyd_reactant.xyz

24
eng= -773.842249
C -0.682785 1.48077 0.419291
O -1.608368 0.67249 1.086088
C -1.909993 -0.603353 0.512146
C -2.632783 -0.496545 -0.824086
C -0.614599 -1.476707 0.478228
O -0.424497 -2.170733 -0.747005
C 0.635387 -0.590153 0.598925
O 1.796113 -1.296073 0.166309
C 0.419324 0.654978 -0.239079
O 1.6441 1.395766 -0.299754
O -1.231195 2.271158 -0.594139
H -2.593796 -1.038732 1.240788
H -0.619039 -2.190057 1.306755
H 0.792425 -0.308398 1.643068
H 0.122311 0.374575 -1.254418
H -0.243094 2.10527 1.206006
H -1.873003 2.881876 -0.215677
H 1.460253 2.246378 -0.716639
H 1.482785 -1.991962 -0.434342
H -1.034724 -2.909346 -0.822863
H -3.008858 -1.474099 -1.13149
H -3.487546 0.168668 -0.7003
H -2.003471 -0.106491 -1.622662
Na 3.482658 0.131592 -0.096616

beta_L_fucose/1.dehyd_ts.xyz

```
24
eng=   -773.773553
C   0.460728   1.313199   -0.738274
O   1.594547   0.810797   -1.04835
C   2.002662   -0.47903   -0.462448
C   2.710925   -0.221927   0.852855
C   0.808386   -1.496934   -0.408328
O   0.683675   -2.080363   0.876463
C   -0.55635   -0.821467   -0.662528
O   -1.625595   -1.609689   -0.173826
C   -0.534696   0.543802   0.072055
O   -1.692372   1.22628   0.180754
O   0.507967   2.695073   0.683934
H   2.722316   -0.8189   -1.205165
H   0.978025   -2.276925   -1.154432
H   -0.727637   -0.687757   -1.734604
H   -0.059232   0.327638   1.049726
H   0.10493   2.066337   -1.436508
H   0.733811   3.606869   0.464851
H   -0.468288   2.634765   0.806025
H   -1.26848   -2.140485   0.554659
H   1.289323   -2.81918   0.981356
H   3.169538   -1.14419   1.211039
H   3.504409   0.507465   0.689684
H   2.034203   0.143977   1.623514
Na   -3.373511   -0.069297   0.188914
```

beta_L_fucose/2.dehyd_reactant.xyz

24
eng=   -773.852231
C   -0.202563   -0.95812   1.212855
O   -1.323385   -0.155927   1.056325
C   -1.658174   0.314967   -0.257159
C   -2.62982   1.46172   -0.066193
C   -0.404099   0.706898   -1.037547
O   0.312405   1.756127   -0.384014
C   0.513808   -0.52795   -1.186314
O   1.875615   -0.102169   -1.212914
C   0.27953   -1.587458   -0.091492
O   -0.706007   -2.452711   -0.604417
O   0.893782   -0.208421   1.748773
H   -2.14366   -0.497883   -0.807191
H   -0.696306   1.049409   -2.034188
H   0.254697   -1.01643   -2.128113
H   1.223007   -2.110113   0.09947
H   -0.466645   -1.734337   1.937395
H   0.641812   0.057825   2.640112
H   -0.845586   -3.1996   -0.014058
H   2.385048   -0.679127   -1.788604
H   -0.237742   2.545109   -0.379902
H   -2.937918   1.870007   -1.031057
H   -3.518539   1.106496   0.454223
H   -2.190167   2.252909   0.546052
Na   2.247331   1.200018   0.566448

beta_L_fucose/2.dehyd_ts.xyz

24
eng= -773.773156
C -1.030189 -0.842861 1.23673
O -1.428159 0.355779 1.149454
C -1.533436 0.961672 -0.213871
C -1.835543 2.421129 0.000984
C -0.181 0.652372 -0.872247
O 0.857091 1.413068 -0.278168
C 0.180829 -0.880172 -0.898345
O 1.435277 -1.166888 -0.615675
C -0.94376 -1.700448 0.079974
O -2.105457 -1.72696 -0.707096
O 2.078752 -0.185747 1.58582
H -2.330784 0.440203 -0.73707
H -0.256624 1.003296 -1.903781
H -0.200873 -1.312703 -1.838455
H -0.512825 -2.677434 0.286346
H -0.630904 -1.105237 2.216633
H 2.443582 -0.630744 2.354296
H -2.708067 -2.403489 -0.381125
H 1.827381 -0.843179 0.860056
H 0.939671 1.190527 0.663451
H -1.911589 2.906285 -0.973791
H -2.787274 2.539521 0.51822
H -1.045488 2.909961 0.568891
Na 2.957487 0.436258 -0.609791

beta_L_fucose/1.ringopen_reactant.xyz

24
eng= -773.85223
C 0.201816 0.958407 1.21281
O 1.322305 0.155654 1.05694
C 1.658171 -0.314505 -0.256596
C 2.630165 -1.460906 -0.065433
C 0.40467 -0.706741 -1.037707
O -0.31178 -1.756187 -0.384626
C -0.513645 0.52783 -1.186696
O -1.875167 0.10134 -1.21367
C -0.279934 1.587405 -0.091818
O 0.705539 2.452906 -0.604463
O -0.895028 0.20919 1.748701
H 2.143599 0.498822 -0.80596
H 0.697426 -1.04895 -2.03429
H -0.254502 1.016377 -2.128454
H -1.223578 2.109847 0.098946
H 0.465784 1.734809 1.937188
H -0.64317 -0.05735 2.639942
H 0.844655 3.199929 -0.014222
H -2.385308 0.68035 -1.786691
H 0.238203 -2.545339 -0.381052
H 3.5185 -1.105522 0.45552
H 2.190454 -2.252434 0.546317
H 2.938882 -1.868762 -1.030278
Na -2.245942 -1.201267 0.566602

beta_L_fucose/1.ringopen_ts.xyz

24
eng= -773.796268
C -0.022986 1.002662 1.276204
O 1.232645 0.121873 1.106126
C 1.671709 -0.218875 -0.242376
C 2.708348 -1.312696 -0.107958
C 0.445117 -0.623031 -1.064769
O -0.191712 -1.754171 -0.463674
C -0.556533 0.558382 -1.172547
O -1.879526 0.033117 -1.199593
C -0.394864 1.616829 -0.063863
O 0.602983 2.508899 -0.509541
O -0.807757 0.008357 1.730004
H 2.106292 0.689997 -0.659642
H 0.771709 -0.891525 -2.072127
H -0.339786 1.081193 -2.107025
H -1.361355 2.117764 0.067598
H 0.277281 1.754385 2.013986
H 0.41909 -0.557137 1.548998
H 0.613152 3.307512 0.026874
H -2.435132 0.583656 -1.758623
H 0.174645 -2.560573 -0.83714
H 3.548799 -0.959599 0.488232
H 2.289162 -2.195156 0.379473
H 3.081838 -1.592559 -1.095167
Na -2.173046 -1.296562 0.631805

beta_L_fucose/2.ringopen_reactant.xyz

24
eng=   -773.85223
C   0.201816   0.958407   1.21281
O   1.322305   0.155653   1.05694
C   1.658171   -0.314505   -0.256596
C   2.630165   -1.460906   -0.065433
C   0.40467   -0.706741   -1.037707
O   -0.31178   -1.756187   -0.384627
C   -0.513644   0.52783   -1.186695
O   -1.875167   0.10134   -1.21367
C   -0.279934   1.587405   -0.091818
O   0.705539   2.452906   -0.604463
O   -0.895028   0.20919   1.7487
H   2.143599   0.498822   -0.80596
H   0.697426   -1.04895   -2.03429
H   -0.254501   1.016378   -2.128454
H   -1.223578   2.109848   0.098946
H   0.465784   1.734809   1.937188
H   -0.64317   -0.057351   2.639941
H   0.844655   3.199929   -0.014222
H   -2.385308   0.680351   -1.786691
H   0.238202   -2.545339   -0.381053
H   2.938882   -1.868762   -1.030278
H   3.518499   -1.105522   0.45552
H   2.190454   -2.252434   0.546317
Na   -2.245942   -1.201267   0.566602

beta_L_fucose/2.ringopen_ts.xyz

```
24
eng=   -773.796267
C   -0.022986   1.002661   1.276204
O   1.232647   0.121878   1.106124
C   1.67171   -0.218872   -0.242379
C   2.708351   -1.312691   -0.10796
C   0.445117   -0.62303   -1.06477
O   -0.191709   -1.75417   -0.463674
C   -0.556535   0.558381   -1.172547
O   -1.879526   0.033112   -1.199597
C   -0.39487   1.616827   -0.063863
O   0.602969   2.508902   -0.509547
O   -0.807754   0.008355   1.730006
H   2.106292   0.690001   -0.659646
H   0.771709   -0.891524   -2.072128
H   -0.339788   1.081193   -2.107025
H   -1.361363   2.117758   0.067601
H   0.277279   1.754386   2.013986
H   0.419096   -0.557136   1.548998
H   0.6132   3.307474   0.026928
H   -2.435135   0.583658   -1.758616
H   0.174661   -2.560572   -0.837126
H   3.081839   -1.592556   -1.095169
H   3.548802   -0.959592   0.488228
H   2.289166   -2.195151   0.379475
Na   -2.173045   -1.296564   0.631808
```

beta_L_fucose/3.ringopen_reactant.xyz

24
eng= -773.84969
C 0.61404 -0.91892 -0.400611
O -0.577572 -1.488291 0.014038
C -1.74133 -0.83861 -0.528876
C -2.949032 -1.587278 -0.011759
C -1.756021 0.648226 -0.14902
O -1.954868 0.752952 1.236956
C -0.425412 1.300168 -0.548762
O -0.291967 2.624533 -0.063081
C 0.698207 0.511919 0.095432
O 1.982928 1.043147 -0.236746
O 1.675377 -1.655308 0.181958
H -1.68658 -0.904047 -1.625226
H -2.574879 1.125036 -0.705883
H -0.308583 1.276292 -1.640448
H 0.560218 0.507454 1.181245
H 0.70999 -0.957481 -1.497935
H 1.3609 -2.557142 0.325862
H 1.908006 2.007044 -0.214031
H -0.755368 3.243371 -0.636015
H -1.929847 1.682632 1.487421
H -2.978747 -1.531514 1.07556
H -3.861907 -1.143099 -0.411824
H -2.901538 -2.63041 -0.322909
Na 3.558586 -0.457039 0.28916

beta_L_fucose/3.ringopen_ts.xyz

24
eng=   -773.785959
C   0.807165   -0.826188   -0.616405
O   -0.505255   -1.479323   -0.40888
C   -1.80779   -0.802564   -0.537357
C   -2.83034   -1.628185   0.206626
C   -1.7233   0.653839   -0.076508
O   -1.779291   0.684674   1.32605
C   -0.437119   1.34361   -0.577303
O   -0.32311   2.651516   -0.047105
C   0.735357   0.564116   -0.017086
O   2.011156   1.145734   -0.258849
O   1.509045   -1.694638   0.141044
H   -1.997481   -0.815351   -1.613539
H   -2.5938   1.16244   -0.514027
H   -0.407697   1.358218   -1.674241
H   0.580622   0.451435   1.061376
H   1.028745   -0.817585   -1.691721
H   0.139369   -2.036984   0.341209
H   1.935024   2.100951   -0.133649
H   -0.790502   3.287512   -0.597411
H   -1.714887   1.600662   1.619857
H   -2.601165   -1.646562   1.271405
H   -3.81525   -1.176371   0.078104
H   -2.857948   -2.642262   -0.191195
Na   3.393706   -0.647053   0.521268

beta_L_fucose/4.ringopen_reactant.xyz

24
eng=   -773.852121
C   0.630394   -0.92644   -0.384674
O   -0.563822   -1.492211   0.040991
C   -1.72189   -0.860647   -0.526815
C   -2.928681   -1.603641   0.00349
C   -1.751418   0.642561   -0.200034
O   -1.916994   0.911093   1.179278
C   -0.41851   1.294327   -0.566561
O   -0.331057   2.643488   -0.185959
C   0.701708   0.506337   0.098838
O   1.979089   1.054202   -0.2301
O   1.696034   -1.652165   0.200218
H   -1.6669   -0.956602   -1.621099
H   -2.550458   1.108779   -0.788038
H   -0.28106   1.264119   -1.652835
H   0.55965   0.506445   1.185249
H   0.716852   -0.975808   -1.482092
H   1.420843   -2.574284   0.280494
H   1.873388   2.015308   -0.282401
H   -0.78616   2.738887   0.661787
H   -2.830074   0.774431   1.446393
H   -3.848621   -1.157389   -0.37992
H   -2.896758   -2.646454   -0.309901
H   -2.936779   -1.575579   1.094376
Na   3.568223   -0.413827   0.297008

beta_L_fucose/4.ringopen_ts.xyz

24
eng=   -773.785434
C   0.827965   -0.835227   -0.600179
O   -0.493147   -1.490487   -0.398591
C   -1.792978   -0.81924   -0.542972
C   -2.814584   -1.633639   0.215644
C   -1.719749   0.655054   -0.119226
O   -1.736622   0.828924   1.285381
C   -0.427625   1.336616   -0.596
O   -0.355812   2.679553   -0.191633
C   0.737974   0.556013   -0.010559
O   2.010068   1.149082   -0.242543
O   1.522529   -1.69888   0.165742
H   -1.98625   -0.855064   -1.618298
H   -2.573162   1.162181   -0.583309
H   -0.382277   1.333206   -1.690002
H   0.576292   0.448565   1.069052
H   1.051758   -0.833092   -1.674952
H   0.141687   -2.040951   0.357285
H   1.903266   2.110547   -0.242209
H   -0.694703   2.736722   0.711649
H   -2.636775   0.807886   1.622194
H   -3.804598   -1.192522   0.080513
H   -2.845947   -2.654518   -0.163788
H   -2.576726   -1.653678   1.280177
Na   3.398662   -0.606572   0.529507

beta_L_fucose/5.ringopen_reactant.xyz

24
eng= -773.841555
C 0.587355 -0.938999 -0.489285
O -0.59158 -1.480298 -0.014527
C -1.762264 -0.804357 -0.508326
C -2.964897 -1.540043 0.038164
C -1.730446 0.669012 -0.098373
O -1.835684 0.741475 1.300334
C -0.424832 1.338957 -0.552384
O -0.430827 2.633869 -0.004643
C 0.740311 0.50744 -0.039333
O 2.025116 1.015696 -0.43853
O 1.659069 -1.675189 0.075488
H -1.747089 -0.85306 -1.60699
H -2.566481 1.182319 -0.588997
H -0.401195 1.373221 -1.653799
H 0.724394 0.530114 1.053735
H 0.637623 -1.014279 -1.589837
H 1.370729 -2.593466 0.156554
H 2.008379 1.258163 -1.372011
H 0.357797 3.12316 -0.254596
H -1.859664 1.670602 1.554595
H -2.955171 -1.503038 1.126604
H -3.8822 -1.071454 -0.321725
H -2.948973 -2.578099 -0.292598
Na 3.452885 -0.436424 0.576482

beta_L_fucose/5.ringopen_ts.xyz

24
eng=   -773.779535
C   0.774412   -0.838752   -0.671128
O   -0.542847   -1.464292   -0.439709
C   -1.82659   -0.74769   -0.538768
C   -2.866343   -1.571685   0.182719
C   -1.690227   0.679256   -0.020974
O   -1.650724   0.639487   1.382615
C   -0.433471   1.38937   -0.561801
O   -0.448635   2.668329   0.023065
C   0.767832   0.577   -0.112733
O   2.049408   1.154173   -0.383817
O   1.467319   -1.700461   0.107741
H   -2.023085   -0.71653   -1.613369
H   -2.568671   1.232403   -0.376161
H   -0.485094   1.452293   -1.659913
H   0.71315   0.497005   0.975613
H   0.987651   -0.878901   -1.749201
H   0.101682   -2.013488   0.32062
H   2.121447   1.389156   -1.317116
H   0.3907   3.117702   -0.11597
H   -1.624005   1.546337   1.709778
H   -2.631977   -1.632664   1.244776
H   -3.839132   -1.089242   0.07513
H   -2.923807   -2.570419   -0.249138
Na   3.311936   -0.69423   0.688355

beta_L_fucose/6.ringopen_reactant.xyz

24
eng= -773.848789
C 0.612593 -0.92847 -0.350857
O -0.597566 -1.450395 0.100316
C -1.732521 -0.825517 -0.537344
C -2.971123 -1.57417 -0.097408
C -1.750056 0.664792 -0.17938
O -2.00666 0.903709 1.190838
C -0.409575 1.311608 -0.548772
O -0.310858 2.652512 -0.143087
C 0.71911 0.515013 0.09207
O 1.997101 1.034775 -0.2722
O 1.667672 -1.657166 0.245884
H -1.606503 -0.912227 -1.625099
H -2.547531 1.158806 -0.74034
H -0.280404 1.298957 -1.637155
H 0.615478 0.540027 1.183786
H 0.681575 -1.012185 -1.446575
H 1.402132 -2.584249 0.299454
H 1.909428 1.997552 -0.329715
H -0.793838 2.747816 0.689566
H -1.707689 0.153843 1.717068
H -3.853823 -1.131452 -0.560831
H -2.901867 -2.619076 -0.39771
H -3.094844 -1.529546 0.985242
Na 3.579073 -0.45048 0.23804

beta_L_fucose/6.ringopen_ts.xyz

24
eng= -773.777696
C 0.798967 -0.803453 -0.435915
O -0.477497 -1.381013 0.027359
C -1.734707 -0.87566 -0.555733
C -2.895429 -1.66724 -0.008249
C -1.734752 0.619101 -0.194462
O -2.041247 0.849947 1.163524
C -0.405409 1.332267 -0.545853
O -0.417944 2.674943 -0.14066
C 0.778983 0.608064 0.094679
O 2.038566 1.184217 -0.218363
O 1.596754 -1.712455 0.146257
H -1.641638 -0.995522 -1.638944
H -2.533012 1.099629 -0.76624
H -0.269014 1.332504 -1.633355
H 0.655454 0.571096 1.185431
H 0.793517 -0.817153 -1.538724
H 0.225863 -2.212977 0.312294
H 1.94963 2.147334 -0.213715
H -0.915354 2.740152 0.685858
H -1.572509 0.224864 1.727476
H -3.821976 -1.263091 -0.419388
H -2.81547 -2.713896 -0.301446
H -2.948934 -1.596979 1.077832
Na 3.496225 -0.610868 0.324653

beta_L_fucose/7.ringopen_reactant.xyz

```
24
eng=   -773.832878
C   0.850824   -0.753179   -0.571157
O   -0.116133   -1.709502   -0.293846
C   -1.483674   -1.278012   -0.082314
C   -1.841585   -1.286011   1.398194
C   -1.719895   0.067845   -0.785509
O   -3.032365   0.525209   -0.61624
C   -0.695274   1.163105   -0.367228
O   -1.169093   2.012426   0.65481
C   0.573046   0.53287   0.177922
O   1.710886   1.392733   0.049837
O   2.095573   -1.273657   -0.119656
H   -2.085964   -2.029312   -0.594305
H   -1.602233   -0.099583   -1.859257
H   -0.436717   1.748929   -1.258574
H   0.431327   0.286585   1.23423
H   0.931202   -0.570732   -1.652714
H   2.017187   -2.236437   -0.12046
H   1.443033   2.280662   0.317497
H   -1.875724   2.565446   0.303861
H   -3.218543   0.605098   0.326032
H   -1.448374   -0.424365   1.941698
H   -2.927559   -1.297627   1.516059
H   -1.45879   -2.198628   1.855679
Na   3.656323   0.282962   0.179233
```

beta_L_fucose/7.ringopen_ts.xyz

24
eng= -773.776896
C 0.996586 -0.593655 -0.702536
O -0.03881 -1.64828 -0.480176
C -1.415151 -1.321048 -0.063315
C -1.607157 -1.381441 1.440553
C -1.745119 0.027271 -0.736041
O -3.064278 0.395046 -0.464067
C -0.75372 1.184967 -0.393568
O -1.221928 2.001067 0.662435
C 0.574864 0.630429 0.081137
O 1.668547 1.539986 -0.015721
O 2.010303 -1.280147 -0.13226
H -2.021209 -2.089368 -0.542053
H -1.694018 -0.140966 -1.814926
H -0.598138 1.783334 -1.298191
H 0.465441 0.325377 1.126672
H 1.075777 -0.419576 -1.782537
H 0.866374 -2.058157 0.079712
H 1.390961 2.396556 0.331345
H -1.855479 2.642088 0.32369
H -3.128345 0.663233 0.46039
H -1.205671 -0.516583 1.969298
H -2.677909 -1.436039 1.646572
H -1.159736 -2.29146 1.844098
Na 3.582678 0.163732 0.30335

beta_L_fucose/8.ringopen_reactant.xyz

24
eng= -773.830424
C -0.896947 1.22697 0.068854
O -0.730435 0.459112 -1.122889
C 0.559516 -0.20809 -1.291113
C 1.706982 0.742272 -1.593259
C 0.776929 -1.160055 -0.096656
O 2.019674 -1.79102 -0.164688
C 0.578553 -0.474617 1.261107
O 1.697514 0.353981 1.461987
C -0.743228 0.309493 1.27915
O -1.841691 -0.617163 1.160387
O -0.023417 2.281591 0.190655
H 0.401243 -0.8279 -2.17776
H 0.030977 -1.961332 -0.162278
H 0.532673 -1.259569 2.027605
H -0.843959 0.9036 2.190663
H -1.938876 1.56956 0.018949
H -0.204973 2.940742 -0.487948
H -2.041436 -1.006367 2.017172
H 1.779317 0.616236 2.383166
H 2.683859 -1.181803 0.182673
H 1.406213 1.42928 -2.386155
H 2.022752 1.308019 -0.720669
H 2.547597 0.154679 -1.965947
Na -2.476308 -0.981182 -0.990143

beta_L_fucose/8.ringopen_ts.xyz

24
eng= -773.746173
C 1.149922 -0.028618 1.252377
O 0.60178 -1.336271 0.146034
C -0.710779 -1.197054 -0.498398
C -1.819239 -1.664812 0.433099
C -0.870873 0.23377 -1.053101
O -2.142203 0.40003 -1.60948
C -0.578584 1.358006 -0.054421
O -1.563743 1.302167 0.944062
C 0.842404 1.26065 0.524851
O 1.80057 1.282407 -0.546451
O 0.486746 -0.523002 2.222743
H -0.686989 -1.868647 -1.362082
H -0.170409 0.361416 -1.886657
H -0.643219 2.296579 -0.620116
H 1.031209 2.079083 1.229367
H 2.227991 -0.265692 1.185675
H 0.386603 -1.4378 1.36107
H 2.01526 2.186084 -0.798003
H -1.653272 2.144045 1.400151
H -2.770107 0.542162 -0.889778
H -1.610195 -2.687346 0.754094
H -1.919004 -1.022436 1.306791
H -2.764108 -1.683772 -0.111189
Na 2.273994 -0.85618 -1.31373
